# High-resolution impedance mapping using electrically activated quantitative phase imaging

**DOI:** 10.1038/s41377-020-00461-x

**Published:** 2021-01-21

**Authors:** Cristina Polonschii, Mihaela Gheorghiu, Sorin David, Szilveszter Gáspár, Sorin Melinte, Hassaan Majeed, Mikhail E. Kandel, Gabriel Popescu, Eugen Gheorghiu

**Affiliations:** 1 International Centre of Biodynamics, 060101 Bucharest, Romania; 2 Institute of Information and Communication Technologies, Electronics and Applied Mathematics, Université Catholique de Louvain, 1348 Louvain-la-Neuve, Belgium; 3 Quantitative Light Imaging Laboratory, Beckman Institute for Advanced Science and Technology, University of Illinois at Urbana-Champaign, Urbana, IL 61801 USA

**Keywords:** Imaging and sensing, Applied optics

## Abstract

Retrieving electrical impedance maps at the nanoscale rapidly via nondestructive inspection with a high signal-to-noise ratio is an unmet need, likely to impact various applications from biomedicine to energy conversion. In this study, we develop a multimodal functional imaging instrument that is characterized by the dual capability of impedance mapping and phase quantitation, high spatial resolution, and low temporal noise. To achieve this, we advance a quantitative phase imaging system, referred to as epi-magnified image spatial spectrum microscopy combined with electrical actuation, to provide complementary maps of the optical path and electrical impedance. We demonstrate our system with high-resolution maps of optical path differences and electrical impedance variations that can distinguish nanosized, semi-transparent, structured coatings involving two materials with relatively similar electrical properties. We map heterogeneous interfaces corresponding to an indium tin oxide layer exposed by holes with diameters as small as ~550 nm in a titanium (dioxide) over-layer deposited on a glass support. We show that electrical modulation during the phase imaging of a macro-electrode is decisive for retrieving electrical impedance distributions with submicron spatial resolution and beyond the limitations of electrode-based technologies (surface or scanning technologies). The findings, which are substantiated by a theoretical model that fits the experimental data very well enable achieving electro-optical maps with high spatial and temporal resolutions. The virtues and limitations of the novel optoelectrochemical method that provides grounds for a wider range of electrically modulated optical methods for measuring the electric field locally are critically discussed.

## Microscopy: phased-in approach puts nanoscale electrical impedance on the map

A microscope capable of real-time, label-free analysis of nanoscale structures and their associated electrical properties could find applications in both biology and materials science. High-resolution microscopy methods that detect variations in the phase of a laser as it passes through samples are helping researchers overcome the limits of traditional optical imaging. In a joint effort, the groups led by Eugen Gheorghiu from the International Centre of Biodynamics in Bucharest, Romania, and by Gabriel Popescu from the University of Illinois at Urbana Champaign have now developed an instrument that combines phase imaging with alternating voltage perturbations to measure a target’s refractive index and electrical impedance maps that can reveal properties including tumors in human tissue, or defects on coating surfaces. The team’s microscope proved capable of distinguishing the impedance contrasts and geometry of different nanostructures on a macroelectrode with submicron resolution.

## Introduction

Optically transparent electrodes decorated with nanometer- to micrometer-scale light-absorbing structures have been increasingly used in the conversion of solar energy into electricity^1^ and in the light-enhanced electrochemical synthesis of important chemicals (e.g., H_2_ from water^2^ and CO from CO_2_^3^). These electrodes also play an important role in biosensor development^4^ and the functional characterization of biological materials from individual living cells to tissue structures^5^.

While there are several methods that investigate the topography of such electrodes with a high spatial resolution (atomic force microscopy (AFM), scanning electron microscopy, etc.), we are still in need of reliable and fast methods that allow functional imaging. Ideally, such techniques will report on the dynamics of electrochemical storage and conversion and perform adequately at various scales down to the single nanoparticle (NP) level^6,7^.

Electrical impedance spectroscopy (EIS) is an established technique used for functional characterization that, when performed across the entire sample, provides an overall quantitative description of the electrical properties of the sample at a certain moment. Studying heterogeneous structures, such as cells, biological tissue, or polydisperse particle mixtures, requires adding spatial resolution to time-lapse impedance assays. Several spatially resolved impedance measurement techniques have been developed and applied to the surface, cell, and particle research, including scanning (electrochemical) probe microscopy and, especially for biological applications, microelectrode arrays. Using extensive nano/microfabrication techniques, EIS measurements managed to a certain extent to bridge the gap between the macro-and microscale electrical properties of inhomogeneous samples. However, this result comes at the price of a reduced time resolution, limited signal-to-noise ratio (SNR), and increased costs.

For example, the spatial resolution of scanning electrochemical microscopy (SECM)^8^, which is a typical method for obtaining electrochemical impedance images of planar samples, was brought into the nanometer range by scanning nanoelectrodes and pipettes. However, the temporal resolution of this technique is still in the several-minutes range, making the method unsuitable for imaging a surface whose properties are rapidly changing^9^. Moreover, images obtained by SECM are always a convolution of the properties of the sample and the (often shifting) properties of the probe^10^.

Scanning Kelvin probe microscopy (SKPM) is another high spatial resolution method that can provide detailed electrochemical information but not directly on the impedance distribution but on the surface potential maps. Similar to SECM, SKPM is characterized by low temporal resolution and is prone to cantilever effects^11^. In addition, SKPM is limited to investigations in a vacuum, air, or electrically insulating, nonpolar liquids^12^.

Optical-based approaches^13^ exploit the sensitive dependency of the refractive index or the surface charge density changes at the electrode–sample interface on the effect of electrochemical processes towards electrochemical impedance microscopy (EIM) concepts. Similar to traditional EIS, optical-based EIS involves applying an AC electrical perturbation onto the electrode–solution interface that leads to a distribution of the electric field modulated by the sample impedance. However, instead of measuring the current that is generated in response to the perturbation, the related variations in the optical properties of the interface are assessed using optical means. Such a route, e.g., surface plasmon resonance microscopy^13^, has been used to obtain electrical impedance images of cells, intracellular processes^14,15^, and protein microarrays^16^. The ability to assess electrochemical reactions with plasmonic-based EIM (P-EIM)^15,17,18^ has also been reported. Accordingly, P-EIM was demonstrated to be an elegant way to measure localized currents and thus localized electrical impedances, despite having a limited SNR (mostly due to speckles), requiring dedicated optical set-ups and substrates with plasmonic features. Label-free, rapid, and high-quality multimodal imaging encompassing electrical impedance remains highly desirable.

Quantitative phase imaging (QPI) has emerged as a powerful, sensitive modality for label-free imaging with nanoscale sensitivity^19^ and, recently, (computational) specificity^20^. QPI enables extracting structural and dynamic information from both opaque and transparent (including live cells) samples without photodamage or photobleaching^21–24^, which is of relevance for investigating interface phenomena^25,26^. The retrieval of dye-free virtual fluorescence images from phase imaging was recently demonstrated in a new microscopy concept, artificial intelligence-enabled, referred to as phase imaging with computational specificity^20^. From the various constructive QPI approaches that have been reported^27–29^, it has been shown that common path approaches offer the benefit of high phase stability suitable for long time-lapse assays. These intrinsically stable methods include diffraction phase microscopy^30^, spatial light interference microscopy^31^, gradient light interference microscopy^32,33^, and magnified image spatial spectrum (MISS) microscopy^34^. The versatility, stability, and quantitative features of QPI are essential for high-quality multimodal imaging encompassing electrical impedance. Moreover, the use of transmission and reflected light microscopy, at the core of QPI assays, has been recently reported as a powerful complement to plasmonic-based microscopy to enable improvement in the spatial resolution of electrochemical sensing^7,35^.

This study presents electrically coupled epi-MISS (el-epi-MISS), which combines MISS in reflection geometry, not reported before, with electrical actuation and plain conductive (but non-plasmonic) probes coupled with a non-faradaic assay to yield high spatial resolution maps of the optical phase (QPI) and electrical impedance. The system combines laser reflected light MISS microscopy^34^ for high-speed QPI with the specific control of the electrode surface charge density changes using low frequency and relatively low magnitude AC electric fields. The dielectric constant of the electrode–liquid interface is thus electrically driven by modulating the double-layer charging in conjunction with the sample electrical impedance fingerprint. Based on this phenomenon, spatial and time processing, we show that el-epi-MISS can be effectively used with QPI and quantitatively related to impedance contrasts at a spatial resolution not accessible before.

Our method is validated by using calibration chips with surfaces designed to provide optical and conductivity contrast at the nanoscale. The spatial resolution is confirmed by AFM and the optical impedance assay by traditional electric impedance assays.

## Results

### Development of a novel set-up enabling epi-MISS and el-epi-MISS

**Fig. 1:**
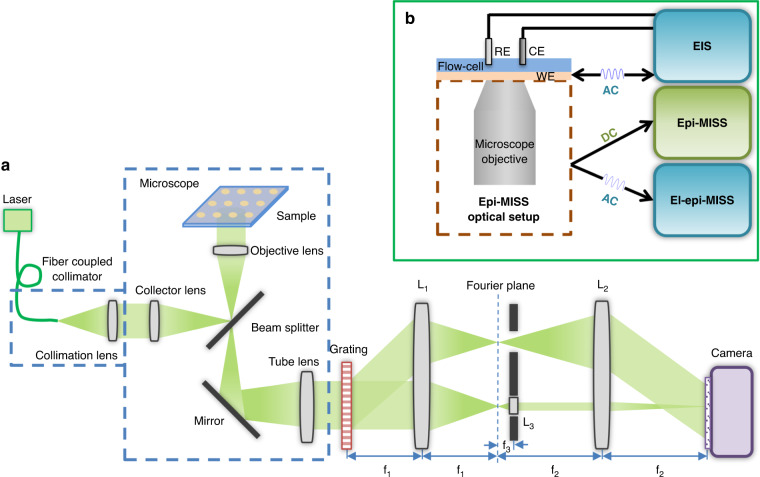
Schematics of the measurement system. **a** The epi-MISS optical set-up based on an inverted microscope, a grating and magnified image spatial spectrum based on a 4-f system with a gradient-index, GRIN, lens (*f*_1_ = 60 mm, *f*_2_ = 150 mm, *f*_3_ = 0.3 mm; *L*_3_ and *L*_2_ form a 4-f system that magnifies the Fourier transform of the zero-order by a factor of 500); **b** Schematic representation of an optoelectrochemical cell with CE, RE, and WE and of the measuring principle

A system enabling electrically actuated, time-lapse epi-MISS imaging was developed around an inverted microscope platform (Fig. 1). The system combines a novel MISS set-up^34^ in reflection (epi) mode (Fig. 1a), an optoelectrochemical cell comprising counter, reference, and working electrodes (CE, RE, and WE), and an electrical AC signal generator (Fig. 1b). This new system yields with high spatial resolution the following quantities: (1) the quantitative phase map (epi-MISS) via the DC component of the electrically actuated time-lapse MISS images and (2) the electrical impedance map of the sample via the distribution of the phase-amplitude oscillation at the frequency of the AC electric field (el-epi-MISS).

### Development of a calibration chip providing electrical and optical contrast at the nanoscale

**Fig. 2:**
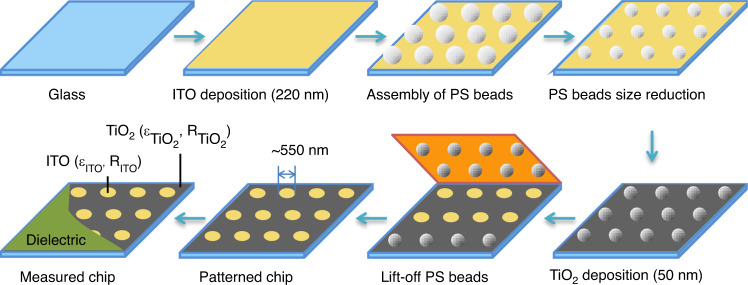
Schematic representation of the nanopatterned calibration chip fabrication steps. The different electrical (relative permittivity, *ε*_*i*_) and optical (reflectance, *R*_*i*_) properties of the two materials, *i* = TiO_2_ and ITO, tailor the optical and electrical contrast of the chip that is included in an electrochemical cell using a dielectric flow cell boundary

A dedicated calibration chip was fabricated to validate the capability of the proposed optoelectrochemical method (Fig. 2). This chip was designed to carry nanometer-sized, disk-shaped areas with optical and electrical properties that are different from those of the background. The chip is integrated as the WE in an electrochemical cell (Fig. 1b) configured with a transparent dielectric coating. The chip, fabricated via sequential deposition, comprises a continuous indium tin oxide (ITO) layer and a perforated titanium dioxide (TiO_2_) upper layer with ~550 nm diameter holes exposing the underneath ITO layer. Note that the TiO_2_ layer has a higher reflectivity than the ITO-exposed surfaces. In terms of electrical contrast, both materials are semiconductors, and their different interfacial electrical properties are expected to provide a contrast that is dependent on the frequency of the applied electrical modulation. Importantly, to highlight the sensitivity of our technique, we constructed the calibration chip to display areas with rather subtle differences in terms of electrical properties; both ITO and TiO_2_ are semiconductors, rather than coarse electrical contrast between, for instance, a dielectric vs. a conductor. For example, a calibration chip featuring only nanostructured, perforated TiO_2_ deposited directly onto glass will have displayed areas with drastic differences in terms of electrical properties. In our set-up, the dielectric reference is offered by the transparent polymer over-layer used to configure the electrochemical cell.

### Optimized protocol to derive 2D maps with topographical (phase) and electrical contrasts

**Fig. 3:**
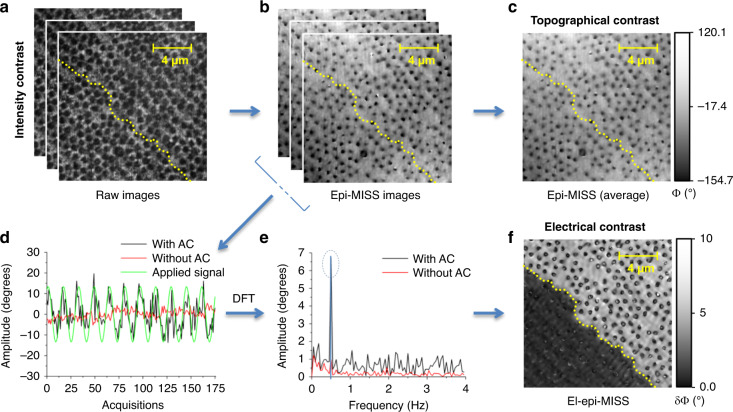
Data processing workflow. **a** Acquired raw images (interferograms) at distinct time points. **b** Reconstructed phase maps (epi-MISS images) at distinct time points. **c** 2D phase map averaged over 176 frames (epi-MISS). **d** Time series of the phase variation (for one point in the field of view) upon AC electrical actuation at 0.5 Hz (black) and without AC (red); the actuation signal is depicted in green. **e** Frequency spectra derived from individual pixels time series in (**d**). **f** 2D map of the optical phase modulation amplitude, δΦ(ω_AC_, *x, y*), derived from the bandpassing signal at approximately 0.5 Hz (el-epi-MISS); the dotted yellow line indicates the polydimethylsiloxane (PDMS) layer delimiting the electrochemical cell; due to its transparency and isolator nature, the PDMS layer is only visible in the map with electrical contrast

As shown in Fig. 3a, each frame acquired over a rectangular area of the chip represents an interferogram that allows the reconstruction of a quantitative phase map Φ(*x*, *y*) via a Hilbert transform method^36^. A heightmap can be further estimated based on the individual pixel quantitative phase information (Fig. 3b), according to:

1 $${\mathrm{h}}(x,y) = \frac{{\Phi \left( {x,y} \right) \cdot \lambda _0}}{{2 \cdot 2{\uppi} \cdot \Delta n}}$$

where *h(x, y)* and Φ(*x*, *y*) are the height and optical phase profiles, respectively, Δ*n* is the refractive index difference (1.33 for our conditions), and *λ*_0_ is the wavelength of the incident light in a vacuum (532 nm for the laser used in our experiments). In reflection, the light travels to the interface and back, requiring an additional factor of 2 in the denominator of Eq. (1) to account for the double pass. The time average over the image sequence Φ(*x*, *y*, *t*) provides the topography of the sample (Fig. 3c).

The dependence of the measured optical phase values, Φ(*x*, *y*, *t*), on the applied AC electric field (Fig. 3d) was demodulated using a discrete Fourier transform (DFT). From the resulting temporal power spectrum bandpassed around the modulation frequency, we precisely extracted the amplitude *δ*Φ(*ω*, *x*_*i*_, *y*_*i*_) of the optical phase modulation as a function of the applied AC electrical signal (Fig. 3e) at every pixel (*x*_*i*_, *y*_*i*_) in the QPI image. The resulting map *δ*Φ(*ω*_*AC*_, *x*, *y*) provides the el-epi-MISS image of interest at the frequency of the AC external electric voltage (Fig. 3f). To minimize the DFT calculation errors, we analyzed the time series over intervals that correspond to an integer number of periods (inverse of the applied AC frequency).

Figure 3c, f illustrates the capability of the proposed approach to reveal both optical and electrical impedance contrast. El-epi-MISS imaging offers a quantitative way to reveal the electrical heterogeneity and contrast of the electrified interfaces, complementing the topographic information. Note that the dielectric over-layer (in green in Fig. 2) is visible only in the el-epi-MISS image (Fig. 3f). Furthermore, high spatial resolution impedance contrast is evident in the area not covered with a dielectric overlay, with distinct spots of higher impedance appearing darker than the surrounding lower impedance regions.

To enable a comparison with the topography map (in Fig. 3c and Supplementary materials, Fig. S3), we expressed the el-epi-MISS image in terms of both the optical phase modulation amplitude *δ*Φ(*ω*_*AC*_, *x*, *y*), which is the actual representation of the electric field modulation according to the Theoretical Section (see Fig. 3f) and the equivalent height modulation $\delta h(x,y) = \frac{{\delta \Phi \left( {\omega _{AC},x,y} \right) \cdot \lambda _0}}{{2 \cdot 2\pi \cdot \Delta n}}$, presented in Supplementary materials, Fig. S3b.

### El-epi-MISS figures of merit

The holes in the TiO_2_ over-layer are clearly visible in the epi-MISS images (Fig. 3b). While this phase contrast is due to the lower reflectivity of the TiO_2_-free regions, the quantitative feature of epi-MISS maps (Fig. 3c) provides a clear estimation of the TiO_2_ layer thickness. Our results indicate an average thickness of ~50 ± 5 nm, which is close to the value targeted by our fabrication design. Figure S3 in the Supplementary materials provides a thorough comparison between these optical measurements and those derived from AFM data.

We validated our spatial resolution by comparing the diameters of the TiO_2_ holes measured by epi-MISS with those measured by AFM. We obtained 470 ± 70 nm, which compares well with the AFM results, of 551 ± 42 nm, as shown in Fig. S3 of the Supplementary materials.

Having a common path geometry^19^, epi-MISS, and el-epi-MISS are characterized by low temporal path-length noise. The temporal noise of el-epi-MISS is given by the standard deviation of the DFT phase-amplitude at the electrical actuation frequency for each pixel and is ~ 0.3 ± 0.1° (Supplementary materials), yielding an SNR of ~30 dB for the electroactive area ($SNR = 10{\mathrm{Log}}[10,({{S}}/{{N}})^2]$). Notably, a similar noise level of ~0.3° is obtained without electrical modulation.

To prove that the el-epi-MISS images (Fig. 3f) effectively reveal the electrical impedance distribution of the structured interface, we used the frequency modulation of the electrical actuation of the el-epi-MISS data in a matching domain with classic EIS measurements of the macro-electrodes.

The dependence of the optical phase oscillation amplitude on the electrical field modulation is described in the Methods section (see “Theoretical model”). This theoretical model allows us to extract the magnitude of the electrical impedance, *Z*_*t*_ (ω_AC_, *x, y*), as a function of the measured δΦ(*ω*_*AC*_, *x, y*), namely:

2 $$Z_t\left( {\omega _{AC},x,y} \right) \approx \frac{{\alpha _{phase}}}{{S \cdot \omega _{AC}}} \cdot \frac{{\delta V_{AC}}}{{\delta \Phi \left( {\omega _{AC},x,y} \right)}}$$

where *α*_*phase*_ is a constant (C^−1^ m^−1^), dependent on the electrode chip structure, *S* is the electrode surface, *ω*_*AC*_ is the angular frequency for the AC field, and δ*V*_*AC*_ is the magnitude of the applied electric potential.

**Fig. 4:**
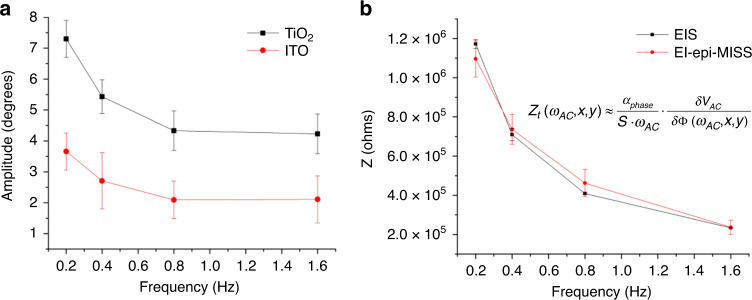
Surface electrical contrasts. **a** The amplitude of the optical phase modulation averaged for multiple specific areas on the surfaces corresponding to holes and the TiO_2_ coating vs. the electrical actuation frequency. **b** Average TiO_2_ impedance measured by both EIS and el-epi-MISS (Z_*t*_(*ω*_*AC*_, *x*, *y*))

El-epi-MISS images were acquired over the same chip area with **δ***V*_*AC*_ kept at the same value of 0.58 V and varying the AC field frequencies between 0.2 Hz and 1.6 Hz. The amplitude of the optical phase variation as a function of the applied AC electrical signal representative of ITO and TiO_2_ materials was derived from averaging specific areas over 3 × 3 pixels (i.e., 10^4^ nm^2^), corresponding to the ITO and TiO_2_ regions (i.e., inside and outside holes). According to Fig. 4a, the amplitude of the phase variation as a function of the applied AC electrical signal reveals quantitative differences between the two materials, indicative of different impedances over the entire applied frequency range.

To quantitatively compare the frequency-dependent el-epi-MISS-derived data to the conventional EIS data (Fig. 4b), the macroscopic impedance of the same TiO_2_-coated electrode was measured by EIS between 0.2 and 1.6 Hz. The modulus of the macroscopic impedance (*Z*) was compared to the el-epi-MISS-derived TiO_2_ impedance since more than 70% of the exposed surface is TiO_2_. Figure 4b shows a good match between the impedance module values derived by the conventional EIS assay and el-epi-MISS. Moreover, as revealed by Fig. 4b, based on Eq. (2), we experimentally derived the value of the linear coefficient *α*_phase_ ~17 C^−1^ m^−1^ (S~1 mm^2^, **δ***V*_*AC*_ = 0.58 V).

**Fig. 5:**
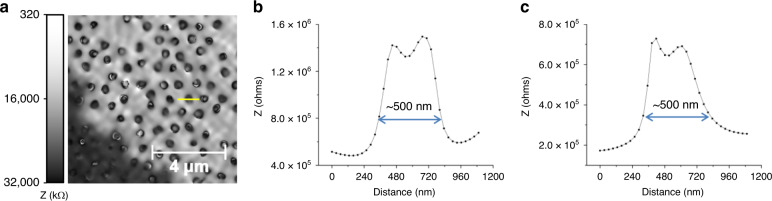
Submicrometric spatial resolution with high electric contrast obtained by el-epi-MISS measurements. **a** El-epi-MISS impedance map at 0.5 Hz. **b** Profile corresponding to the yellow line and indicating a generic, individual hole in the TiO_2_ layer. **c** The same profile corresponding to an individual hole in the TiO_2_ layer at 1.6 Hz

Based on Eq. (2), an impedance map of the nanopatterned surface is derived (Fig. 5a), and the profiles corresponding to holes in the TiO_2_ layer an ~500 nm diameter (Fig. 5b), irrespective of the analyzed frequency (Fig. 5c). A thorough analysis of the hole diameters in el-epi-MISS images (see Supplementary materials Fig. S3b) highlights a 513 ± 48 nm average diameter, which compares well with the diameter revealed by AFM measurements (551 ± 42 nm).

## Discussion

Optical imaging with electrical field modulation contains quantitative information on both the double-layer charging and faradaic currents and can be used to perform electrochemical assays, e.g., impedance spectroscopy and voltammetry (cyclic and square-wave), in an analogous way to traditional electrochemical methods^15,37,38^. This finding was previously demonstrated with plasmonic chips and P-EIM assays. Due to the inherently restricted signal-to-noise ratio and the limitations that are typically associated with plasmonic chips and P-EIM^38–40^ assays e.g., the dependency on the wavelength, the polarization and angle of incidence of the illumination, the orientation-dependent propagation length restricting the spatial resolution, alternative high-resolution (microelectrode free) impedance maps of model nanostructured interfaces have been constantly pursued. Our approach fulfills this need and demonstrates improved optical and electrical resolution based on the spatial and temporal FT processing algorithms associated with a multimodal imaging system. Our results are consistent with previous reports showing that the surface charge density modulates optical information issued by other imaging methods^7^ and rely on a modular QPI format to decouple the geometrical (thickness) and electro-optical (complex refractive index) variations.

Progressing from the limitations associated with plasmonic chips^13,18^ and the use of chemical mediators^18^, we exploit QPI and ITO surfaces for the mediator-free, high-resolution mapping of the electrical impedance and of the optical path differences of reproducible patterns on top of transparent (semi)conductive surfaces.

Using electrically modulated reflection-geometry MISS (el-epi-MISS), we demonstrate the real-time assessment of QPI and high spatial resolution magnitude impedance maps of model nanostructured interfaces. Thus, optically transparent chips with rather subtle differences in terms of electrical properties are mapped with submicrometer lateral resolution. In addition, we describe the theoretical formalism that relates the electrical modulation of the refractive index to the variation in the reflected field phase.

Our el-epi-MISS approach was validated by classic EIS measurements. Notably, the amplitude of the optical phase oscillation at the electrical actuation frequency provides a consistent response with conventional measurements (EIS) and with our theoretical model for the tested frequency range.

The proposed method presents the following capabilities:

- Assessment of inhomogeneous electrical properties with high spatial and temporal resolution. As such, electrical contrasts associated with material inhomogeneity (both electrical and structural as well as the presence of dielectric nanoscale features) are quantitatively imaged with limited processing requirements at high speed and at high resolution. Our approach outperforms high-end SECM instruments in terms of speed and P-EIM in terms of spatial resolution. Moreover, QPI eliminates several strict requirements for P-EIS microscopy, such as the dependence on the wavelength, the polarization, and the angle of incidence. This allows us to obtain an isotropic spatial resolution, independent of the propagation length in P-EIS.
- The electrical and optical properties are analyzed using common sensing surfaces (such as transparent conductive electrodes that are widely utilized as biointerfaces and as light-harvesting elements) and not dedicated chips as for P-EIS. P-EIS requires thin films (mostly made of Au) with a well-defined thickness of approximately 50 nm. However, QPI can be carried out on a larger variety of materials and thicknesses, as demonstrated with the ITO conductive thin film used as WE.
- El-epi-MISS renders accurate quantitative phase/topographic maps of the specimen tested. The hole dimensions provided by AFM (Supplementary materials Fig. S3) are consistent with the values provided by the el-epi-MISS assays. Interestingly, the dimension of the el-epi-MISS-derived average hole diameter is closer to the AFM results, highlighting an improvement in the spatial sensitivity of the electrical assay.
- The proposed approach adds a whole new capability to quantitative phase microscopy assays, for example, impedance mapping beyond the spatiotemporal resolution provided by micro/nanoelectrodes as scanning probes or microelectrode arrays.
- El-epi-MISS microscopy does not involve intricate developments; it can be implemented as an upgrade module to an existing inverted microscope, converting it into a powerful multimodal imaging instrument. Unlike P-EIS, el-epi-MISS has a much simpler optical set-up, without the need for a specific angle of incidence or advanced optics.

Thus, we anticipate that el-epi-MISS will be adopted broadly for both materials and life sciences (including biomedical) applications.

The formalism discussed in this work is general; it encompasses the core of the electro-optical phenomena and applies as well to other electro-optical methods, e.g., plasmonics^13,41^, with particular values for the respective linear coefficient (i.e., *α*_phase_), and in principle to impedance assays in any radio-frequency domain.

However, certain limitations that apply to both el-epi-MISS and P-EIM are worth mentioning. Specifically, the frequency of the AC field, at the denominator of the cause-effect relation, Eq. (2) (as highlighted in the present study for non-faradaic approaches), is a limiting factor. It restricts the applicability of electro-optical assays to frequencies below ~hundreds of kHz, even if, now available^42^, cameras with megahertz frame rates are deployed.

## Materials and methods

### The el-epi-MISS system

In MISS microscopy, the reference field for QPI is generated using a GRIN lens (350 µm diameter, an effective focal length of 300 µm) to magnify the Fourier transform of the zero-order to the point where the DC component fills the camera sensor^34^. MISS is characterized by low spatiotemporal path-length noise (0.6 nm temporal noise and 2.8 nm spatial noise floor)^36^. Due to the more efficient collection of the reference order and highly stable low noise statistics, MISS is ideal for high-throughput QPI investigations, such as dynamic light scattering and cellular membrane mechanics^34^ assays.

The light emitted by the laser diode laser (LMX-532L-100-COL-PP, Oxxius, France) at 532 nm was fiber-coupled, collimated, and used as an epi-illumination source for an inverted microscope (Axio Observer Z1, Zeiss, Oberkochen, Germany). All imaging was carried out using a 100× oil immersion objective (1.46 NA, WD = 0.11 mm). The nanostructured slide was illuminated from the bottom, and the reflection image plane exiting the microscope was relayed to an EMCCD camera (iXon^EM^ + 885, Andor, Belfast, UK) using a 4f system based on lenses *L*_1_ and *L*_2_ (Fig. 1a). A diffraction grating (Edmund Optics, New Jersey, USA; 110 lp/mm) was placed precisely at the image plane outside the microscope to separate the image field into multiple orders, each containing complete information about the object. At the Fourier plane of *L*_1_, the zeroth diffraction order was passed through a GRIN lens *L*_3_ (Edmund Optics), while the first diffraction order passed through unaffected. In combination with *L*_2_, lens *L*_3_ formed a 4f system that produces a highly magnified image of the zeroth-order Fourier spectrum at the camera plane^34^.

For the el-epi-MISS measurements, the time series of variable length (between 200 and 400 frames) of the reflectivity interferograms of magnified images of actuated nanostructured surfaces were acquired at up to 21.3 frames per second when driven by sinusoidal fields with frequencies between 0.2 and 1.6 Hz (0.2, 0.4, 0.5, 0.8, 1.6 Hz). The system was actuated by applying a signal of 0.58 V, suitable to drive stable oscillations of the optical intensity.

When illuminated with laser light and imaged in the reflection mode using the set-up in Fig. 1, each interferogram was processed based on the spatial Fourier transform^36^ to reconstruct the phase map of the optical contrast of the chip structure.

The dependence of the phase map Φ(*x*, *y*) on the applied electric actuation (*V*_*AC*_ amplitude and frequency) for each pixel was reflected in the time series of the quantitative phase images Φ(*t*, *x*, *y*) that were analyzed by a DFT performed on each pixel of the phase map Φ(*x*, *y*) in the time domain.

The resulting map of the amplitude of the oscillatory response of the quantitative phase values *δ*Φ(*ω*_*AC*_, *x*, *y*) upon actuation, derived from DFT at the applied frequency, provides the el-epi-MISS image.

### Signal generation and conventional electrical measurements

An impedance analyzer (Solartron 1260, Hampshire, UK) and a potentiostat (CellTest 1470E, Solartron) were used to impose AC actuation over the microfluidic electrochemical cell assessed using both el-epi-MISS and conventional EIS measurements. The electrochemical cell consists of a three-electrode system: miniaturized Ag/AgCl, 3M KCl, (World Precision Instruments, Florida, USA) as the RE, Pt wire as the CE, and the nanopatterned surface as the WE, exposed to the electrolyte in a PDMS gasket (Fig. 1b).

### Nanopatterned chip

Figure 2 presents the fabrication steps of the nanopatterned chips. First, an ITO layer was deposited on a glass substrate by physical vapor deposition. Then, nanopatterning was achieved via colloidal lithography. This operation requires the self-assembly of close-packed polystyrene (PS) spheres 980 nm in diameter on the ITO substrate. The PS spheres were further etched with reactive ion etching to the desired size, representing the final diameter (~550 nm). Subsequently, TiO_2_ was deposited on the PS-decorated ITO surface. The nanopatterned chip was obtained by lift-off of the PS spheres with adhesive tape, followed by a cleaning step. The detailed protocol is presented in the Supplementary materials.

### Materials

All electrical measurements were performed in a phosphate-buffered saline solution (pH 7.4) containing 137 mM NaCl, 10 mM phosphate, and 2.7 mM KCl as the running buffer. Ultrapure water (Merck Millipore, Guyancourt, France) was used throughout the preparations. All chemical reagents were purchased from Sigma Aldrich (Missouri, USA), were of analytical grade, and were used without further purification.

### Theoretical model

This section and the Supplementary materials substantiate the background to measure the electric field locally with an optical phase method.

The physical and chemical phenomena that occur when an electric field is applied at the electrode–liquid interface have been extensively discussed in the literature^43,44^.

Different mechanisms take place at this interface and can be responsible for altering the refractive index of the interface and the optical phase values, namely, the changes in the Helmholtz double-layer, electronic density, and oxidation of the electrode film. These phenomena depend on both the electrode material and the solution in contact with the electrode as well as on the amplitude of the applied potential. According to Gouy–Chapman theory, the surface charge density at the electrode–electrolyte interface for symmetric electrolyte is equal to^45^:

3 $$\sigma = \left( {8kT \cdot \varepsilon _l\varepsilon _0 \cdot n} \right)^{1/2}\sinh \frac{{z \cdot e \cdot \left( {\varphi _0 - \varphi _s} \right)}}{{2kT}}$$

where *k* is the Boltzmann constant, *T* is the absolute temperature, *ε*_*l*_ is the dielectric constant of the electrolyte solution, *ε*_0_ is the permittivity of vacuum, *n* is the bulk concentration of charges, *z* is the valence of the electrolyte, *e* is the elementary electric charge, *φ*_0_ is the electrostatic potential at the electrode–electrolyte interface, and *φ*_*s*_ is the electrostatic potential at the outer Helmholtz layer.

Based on the Drude–Lorentz model^46–48^, one can derive the dependence of the components of the dielectric constant of the thin layer *ε*_*tl*_ of the solid facing the electrolyte (with the thickness equal to the penetration depth of the quasi-static electromagnetic field) on the surface charge distribution, as well as the related variations due to an AC electric field^49^:

4 $$\delta \varepsilon ^{\prime}_{tl}\left( {\omega _{AC},x,y} \right) = - \left( {\varepsilon ^{\prime}_{tl} - 1} \right) \cdot \frac{{\delta \sigma \left( {\omega _{AC},x,y} \right)}}{{n_e \cdot e \cdot d_{tl}}}\\\delta \varepsilon ^{\prime\prime}_{tl}\left( {\omega _{AC},x,y} \right) = \varepsilon ^{\prime\prime}_{tl}\frac{{\delta \sigma \left( {\omega _{AC},x,y} \right)}}{{n_e \cdot e \cdot d_{tl}}}$$

where *d*_*tl*_ is the penetration depth of the electric field (the Thomas–Fermi screening length) within the electrode at the electrode–liquid interface, *ε*_tl_ ($\varepsilon _{tl} = \varepsilon ^{\prime}_{tl} + j\varepsilon ^{\prime\prime}_{tl}$) is the free-electron contribution to the dielectric constant of the electrode, and *n*_*e*_ is the free-electron density of the bulk solid. Corresponding to the same material, *ε*_*tl*_ differs from *ε*_*el*_ due to the biasing effect of the DC value of the electrode potential, which is additionally modulated by the local AC field.

According to equations (s1) and (s2) in the Supplementary materials, the AC electrically modulated surface charge distribution at the electrode–liquid interface is:

5 $$\delta \sigma \left( {\omega _{AC},x,y} \right) \approx - \varepsilon _l\varepsilon _0\delta \frac{{\partial \mathop {\varphi }\nolimits_l \left( {\omega _{AC},x,y,z \to {\mathrm{0}}} \right)}}{{\partial \;{\mathrm{z}}}}$$

where *φ*_*l*_(*ω*_*AC*_, *x*, *y*, *z*→0) is the local AC electric potential in liquid at the electrode–liquid interface and *ε*_*l*_ is the relative permittivity of the liquid.

Since our assay involves non-faradaic currents^13,50^ (low applied potentials), the variation in the surface charge density is related to sample impedance (for derivation, see equations (s3)–(s5)) via:

6 $$\delta \sigma \left( {\omega _{AC},x,y} \right) = \frac{{\delta V_{AC}}}{{\omega _{AC} \cdot S \cdot Z_t\left( {\omega _{AC},x,y} \right)}}$$

Consequently, one obtains:

7 $$\begin{array}{l}\delta \varepsilon ^{\prime}_{tl}\left( {\omega _{AC},x,y}\,\,\, \right) = - \frac{{\left( {\varepsilon ^{\prime}_{tl} - 1} \right)}}{{e \cdot n_e \cdot d_{tl}}} \cdot \frac{{\delta V_{AC}}}{{\omega _{AC} \cdot S \cdot Z_t\left( {\omega _{AC},x,y} \right)}}\\\delta \varepsilon ^{\prime\prime}_{tl}\left( {\omega _{AC},x,y} \right)\ = \frac{{\varepsilon ^{\prime\prime}_{tl}}}{{e \cdot n_e \cdot d_{tl}}} \cdot \frac{{\delta V_{AC}}}{{\omega _{AC} \cdot S \cdot Z_t\left( {\omega _{AC},x,y} \right)}}\end{array}$$

**Fig. 6:**
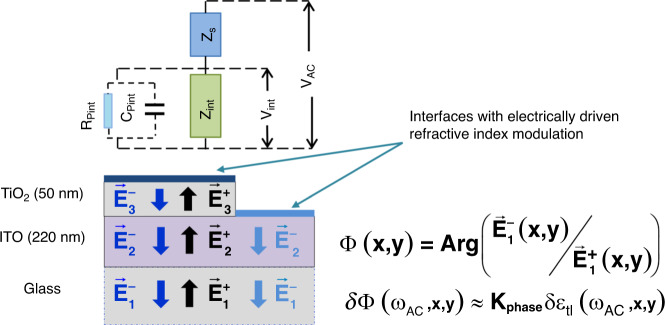
Schematic representation of the equivalent electrical circuit. Phase variation is linearly dependent on the relative permittivity shift, driven by the (local) electric field, of the liquid–sensor interface

To relate the electrical structure of the sample to the 2D phase map when applying an AC electric field in an epi-MISS set-up, one has to connect the impedance formalism above to the multilayered sensing chip in the context of the general formalism based on Fresnel equations to derive the 2D distribution of the phase amplitudes as a function of the electrical impedance fingerprint of the sample and the parameters of the chip (as illustrated in Fig. 6).

Indeed, based on Fresnel equations, we derive the variation in the phase difference between the incident and reflected light (*Φ*(*x*, *y*) in equation (s7), Supplementary materials), modulated by the electrically active upper-most interface, also dependent on the structure of the sensing chip (*ε*_*i*_*, d*_*i*_):

8 $$\delta \Phi \left( {\omega _{AC},x,y} \right) = \delta \Phi \left[ {\delta \varepsilon _{tl},Z_t\left( {\omega _{AC},x,y} \right),\varepsilon _i,d_i} \right]$$

The phase variation is linearly dependent (in a first approximation), via *K*_phase_, on the relative permittivity shift, δ*ε*_*tl*_ driven by the (local) electric field, of the liquid–sensor interface. For a given structure of the sensing chip (with known parameters *ε*_*i*_ and *d*_*i*_), the variation in the phase magnitude relates to δ*ε*_*tl*_ via:

9 $$\delta \Phi \left( {\omega _{AC},x,y} \right) \approx K_{phase}\delta \varepsilon _{tl}\left( {\omega _{AC},x,y} \right)$$

Consequently, since the highest impact is given by ε′_tl_,

10 $$\delta \Phi \left( {\omega _{AC},x,y} \right) \approx K_{phase} \cdot \frac{{\left( {\varepsilon^{\prime}_{tl} - 1} \right)}}{{e \cdot n_e \cdot d_{tl}}}\frac{{\delta V_{AC}}}{{S \cdot \omega _{AC}}}\frac{1}{{Z_t\left( {\omega _{AC},x,y} \right)}}$$

By denoting *α*_phase_ as:

11 $$\alpha _{phase} = K_{phase} \cdot \frac{{\left( {\varepsilon^{\prime}_{tl} - 1} \right)}}{{e \cdot n_e \cdot d_{tl}}}$$

one obtains the dependence of the variation in the optical phase-amplitude on the magnitude of the electrical impedance of the sample (similar to Eq. (2)):

12 $$\delta \Phi \left( {\omega _{AC},x,y} \right) \approx \alpha _{phase}\frac{{\delta V_{AC}}}{{S \cdot \omega _{AC}}}\frac{1}{{Z_t\left( {\omega _{AC},x,y} \right)}}$$

Combining Eqs. (6) and (11), one relates the phase to the AC variation of the surface charge density:

13 $$\delta \Phi \left( {\omega _{AC},x,y} \right) \approx \alpha _{phase}\delta \sigma \left( {\omega _{AC},x,y} \right)$$

Both the theoretical simulations and the experimental results reveal a quasi-linear dependency of the phase-amplitude, *δΦ*, on the excitation, with *δV*_*AC*_ yielding a similar value of the linear coefficient *α*_phase_, ~17 C^−1^ m^−1^.

Notably, the simulation reveals that the ratio *K*_phase_/*d*_*tl*_ does not depend on *d*_*tl*_ in the range of 1 nm ÷ 0.1 nm; therefore, assuming the precise value of the Thomas–Fermi screening length is not critical for deriving *α*_phase_.

The relations in Eq. (7) encompass the core of the electro-optical phenomena concerning non-faradaic approaches. They generically relate the modulation of the dielectric constant of the thin layer of the electrode facing the electrolyte to the sample impedance and the parameters of the applied electric field. As a limiting factor of this cause-effect relation, the frequency of the AC field at the denominator restricts the applicability of electro-optical assays to frequencies below ~100 kHz, even though cameras with megahertz frame rates became available^42^.

Moreover, Eqs. (12) and (13) apply to other electro-optical methods, e.g., involving plasmonics^13,41^, with particular values for the respective linear coefficient.

## Supplementary information

Supplementary materials

## Data Availability

The data that support the findings of this study are available from the corresponding authors upon reasonable request.
