# Supplementary material for: High-resolution impedance mapping using electrically activated quantitative phase imaging
Source: Light Sci Appl. 2021 Jan 21;10:20. doi: 10.1038/s41377-020-00461-x (PMC7820407; doi:10.1038/s41377-020-00461-x)
Supplement: Supplementary file 1 — Supplementary materials [file 41377_2020_461_MOESM1_ESM.docx]

**High-resolution impedance mapping using electrically activated quantitative phase imaging**

Cristina Polonschii^1^, Mihaela Gheorghiu^1^, Sorin David^1^, Szilveszter Gáspár^1^, Sorin Melinte^2^, Hassaan Majeed^3^, Mikhail E. Kandel^3^, Gabriel Popescu^3^*, Eugen Gheorghiu^1^*

^1^ International Centre of Biodynamics, 060101, Bucharest, Romania

^2^ Institute of Information and Communication Technologies, Electronics and Applied Mathematics, Université catholique de Louvain, 1348, Louvain-la-Neuve, Belgium

^3^ Quantitative Light Imaging Laboratory, Beckman Institute for Advanced Science and Technology, University of Illinois at Urbana-Champaign, Urbana, Illinois 61801, USA

### Supplementary materials

**I. Impedance formalism**

We consider the simplified equivalent circuit model of the electrode-electrolyte interface depicted in **Figure** **S1**. When applying a low amplitude AC electric field (yielding non-faradaic currents), the boundary condition for the normal component of the electric displacement at the interface provides the related variation of the surface charge density, σ:

(s1)

(s2)

where ϕ_l_(x, y, z→0) and ϕ_el_(x, y, z→0) represent the local AC electric potential in liquid, and in electrode, respectively, at the electrode-liquid interface, and ε_el_ and ε_l_ represent the relative permittivities of the two media. The electric field in metallic electrodes is negligible; however, the electric field in semiconductors (*e.g.*, in ITO and TiO_2_ layers), while less than the one in liquid, is non negligible thus renders the approximation stated in equation s2.

For the electronic structure of Ti we assumed well-known parameters, namely density of states and Fermi energy, and inferred a value of 0.55 x 10^28^ m^-3^ for the electron density^1,2^.

**
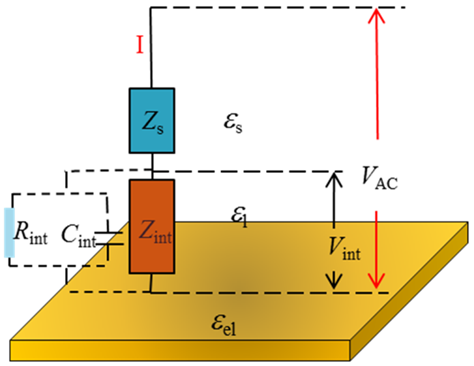
**

**Figure S1.** Schematic representation of the equivalent circuit model of the electrode-electrolyte interface

Rewriting equation s2 in relation to interfacial capacitance, *C*_int_:

$\text{ }\delta\sigma\left(\omega_{AC},x,y\right)\text{=}\frac{C_{\text{int}}\left(x,y\right)\cdot\delta V_{\text{int}}\left(\omega_{AC}\right)}{S}$ (s3)

Assuming the non-faradaic condition ($\left|\frac{1}{\omega_{AC}C_{\text{int}}}\right|<<R_{\mathrm{int}}$):

$\delta\sigma\left(\omega_{AC},x,y\right)\text{=}\frac{\delta V_{\text{int}}\left(\omega_{AC}\right)}{S\cdot\omega_{AC}\cdot Z_{\text{int}}\left(\omega_{AC},x,y\right)}$ (s4)

According to the equivalent circuit model in **Figure S1,** we introduce the total impedance,$Z_{t}\left(\omega_{AC}\right)=Z_{\mathrm{int}}\left(\omega_{AC}\right)+Z_{s}\left(\omega_{AC}\right)$:

$\delta V_{\mathrm{int}}\left(\omega_{AC}\right)\text{=}\frac{Z_{\mathrm{int}}\left(\omega_{AC}\right)}{Z_{t}\left(\omega_{AC}\right)}\delta V_{AC}$ (s5)

From equations s4 and s5 one obtains^3^:

$\delta\sigma\left(\omega_{AC},x,y\right)\text{=}\frac{\delta V_{AC}}{\omega_{AC}\cdot S\cdot Z_{t}\left(\omega_{AC},x,y\right)}$ (s6)

that relates the change of the surface charge density to the variation of the applied field and the total impedance of the sample for the applied frequency.

**II. Simulation of the phase amplitude variation, *δΦ*, dependency on $\delta\sigma\left(\omega_{AC},x,y\right)$ induced by an external excitation**

We consider a multilayered chip (electrode) structure (see **Figure 6** main text) consisting of: the first semi-infinite layer of glass with dielectric permittivity *ε*_p_ = 2.304, a second layer of ITO with thickness d_ITO_ = 220 nm and complex permittivity, *ε*_ITO_ = 4.13 + j 0.031 and, when present, a third layer, Ti (eventually TiO_2_), with thickness d_TiO2_ = 50 nm, and dielectric permittivity ε_TiO2_ = 6. The solution, that is the upper medium, has a dielectric permittivity ε_s_ = 1.8. The interface between the electrode and the solution is modeled by considering a material specific (*i.e.,* either ITO or Ti) intermediary thin layer, whose electronic charge density is modulated by the local surface charge density: a layer of thickness d_tl_ ~1 nm equal to the penetration depth of the quasi-static electromagnetic field. Accordingly, in the regions where ITO film is coated, the thin layer with d_tl_ ~1 nm thickness is particular for Ti (progressing to TiO_2_) and has ε_Titl_ = 5.5 permittivity.

Whereas the actual composition of the Ti over-layer deposited on ITO is not known, we considered for the numerical computations a structure with upper layers composed of a mixture of Ti and TiO_2_. The rationale behind this approximation is the following: vacuum-deposited titanium is inherently associated with the development of an oxide film when exposed to (even limited amounts of) air. The thickness of the oxide film formed on titanium was reported to be about 1.7 nm thick after 2 h exposure to air at room temperature and reach about 3.5 nm after longer (40 to 50 days) exposure in the same conditions^4^. An even thicker oxide film (5-10 nm) was reported to occur on heat sterilized titanium samples owing to the elevated temperatures involved^5,6^. The TiO_2_ formed preferentially on titanium at temperatures under 200 °C^7^.

Considering the Fresnel equations and the chip structure one derives the phase:

$\Phi\left(\omega_{AC},x,y\right)=Arg\left(\frac{\vec{E}_{1}^{-}\left(\omega_{AC},x,y\right)}{\vec{E}_{1}^{+}\left(x,y\right)}\right)\text{ }$ (s7)

allowing for the theoretical simulation of the phase amplitude variation, *δ*Φ, dependency on surface charge density variation $\delta\sigma\left(\omega_{AC},x,y\right)$ induced by an external excitation.


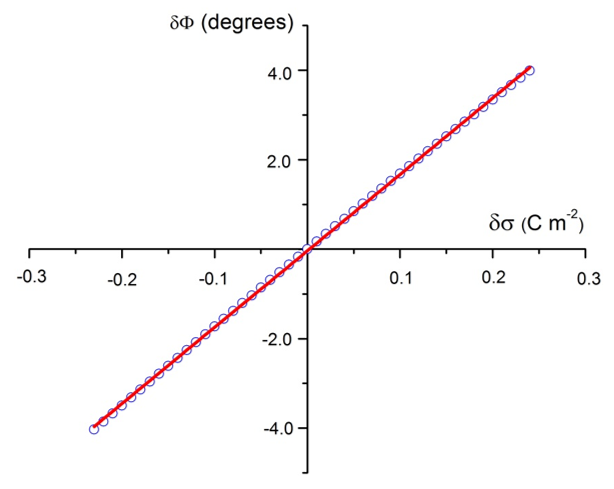


**Figure S2.** The quasi-linear dependency of phase amplitude, *δΦ*, in degrees, on excitation induced change of the surface charge density $\delta\sigma\left(\omega_{AC},x,y\right)$; with red, the linear fit

The linear dependency of phase amplitude shown in **Figure S2**, according to equation (13 main text), yields the theoretical value of *α*_phase_ =17, in good agreement with the experimental data measured by el-epi-MISS and by a conventional EIS assay (in **Figure 4b**, main text).

**III. Preparation of the nanopatterned chips**

Before colloidal lithography, step in the fabrication of the nanopatterned calibration chip (according to Figure 2 main text), the ITO-coated slides were soaked in an acetone bath at 60 °C for 10 minutes under ultrasonic cleaning, then thoroughly rinsed with isopropanol and blown dry under nitrogen. The substrates were rendered hydrophilic after 2 minutes of oxygen plasma treatment, which was carried out by introducing 20 sccm oxygen gas flow into the reaction chamber of a plasma cleaner (K1050X, Quorum Technologies, Lewes, UK), under a pressure of 0.6 mbar with 30 W radio-frequency power.

Polystyrene (PS) microspheres were purchased from Microparticles GmbH (Berlin, Germany) as 5% w/w, monodispersed aqueous suspensions. The PS particles were applied at the water-air interface by drop casting 50 µl suspension on a tilted glass slide that was partially immersed in deionized water. The PS colloidal suspension immediately spread outwards over water surface due to the surface tension gradient between ethanol-containing colloidal suspension and the deionized water. The floating particles began to crystallize and form hexagonally closed-packed, two-dimensional colloidal crystals on the water-air interface. Then, 10 µl surfactant Triton X-100 (Sigma Aldrich, USA), pre-diluted to 1% v/v with water, was added to the interface to consolidate the particle monolayer. Transferring the floating PS beads from water-air interface to the ITO coated substrate was conducted by first immersing the substrate in water, then positioning the sample right under the desired region of the monolayer, and lifting it up to pick the self-assembled particles. Finally, the transferred PS microsphere monolayer was dried at room temperature for 4h.

To reduce the size of the PS microspheres on the ITO coated glass microslides, assembled colloidal arrays were etched by reactive ion etching in an Oxford Plasmalab 100 system (Oxford Instruments, Abingdon, UK), where an oxygen plasma was activated from a 30 sccm oxygen gas flow at 30 W forward power under a 5 mTorr vacuum and a DC bias of 163 V. The temperature of the sample holder was kept constant at 15 °C. After 11 minutes etching, the PS microspheres with initial diameters of 980 nm were shrunk to ~550 nm. Final hole diameters are ±10% of the average value.

Titanium was then coated onto the chips by physical vapor deposition yielding a thin, 50 nm thickness, film of titanium oxide. The rate of deposition was kept constant below 1 Å s^-1^ to ensure a good quality of the film. After physical vapor deposition, the PS microspheres were removed by dissolving them in chloroform for 1 h, followed by ultrasonic cleaning in fresh chloroform for another 5 minutes, and thoroughly rinsing with isopropyl alcohol and blow drying with nitrogen.

**IV. Complementary AFM characterization of the nanopatterned chip and estimation of TiO_2_ holes diameter**

A Nanowizard II AFM instrument (JPK Instruments AG, Berlin, Germany), operating in intermittent contact in air, with an ARROW-NCPt cantilever (resonant frequency of 285 kHz, force constant of 42 N m^-1^, from NanoAndMore GmbH, Wetzlar, Germany) was used to reveal the topography of the nanostructured electrode, complementary to el-epi-MISS microscopy. Slow (~0.3 Hz) line rates and relatively high setpoints (~450 mV) were used during scans.

The epi-MISS, el-epi-MISS and AFM images were analyzed as follows: using a threshold, binary images are generated and then a “find circles” algorithm (LabView, National Instruments, Texas, USA) is used to separate circular particles and classify them based on their radius, surface area, and perimeter. Starting from a binary image, the algorithm constructs and uses a Danielsson distance map^8^ to determine the radius of each particle. As seen in **Figure S3**, epi-MISS and el-epi-MISS underestimate the dimension of the “particles” associated with holes, possibly due to the diffraction limit which is on the order of λ/(2 NA) ~182 nm. Another factor to be considered is the fringes contrast for the acquired interferograms used to reconstruct the topographical maps. **Figure 3a** in the main text shows such an interferogram, with TiO_2_ holes appearing darker than the rest, and where the fringes contrast is poorer, therefore yielding higher reconstruction errors. Interestingly, el-epi-MISS yields hole diameter values closer to the AFM results, the electrical contrast improving the sensitivity of standard epi-MISS images.

**Figure S3.** Surface characterization of the ITO-TiO_2_ nanostructured chip by: **a.** epi-MISS; **b.** el-epi-MISS; **c.** AFM; ROIs used to calculate the holes diameter are superimposed; In comparison with AFM, hole dimension given by epi-MISS and el-epi-MISS is underestimated.

**V. Characterizing the noise in the el-epi-MISS system**

To assess both the signal and the temporal noise of the el-epi-MISS system, DFT was applied on 16 time series each of 128 phase images. The signal is given by the averaged amplitude of the phase modulation at the electrical actuation frequency (0.5 Hz) and the corresponding noise by the related standard deviation. For a 20×20 pixels area in the electro-active layer (on TiO_2_) there is a 0.27 ° standard deviation of the mean DFT derived phase amplitude values at the electrical actuation frequency, centered around 6.7 °, that compares well with the single pixel values. According to **Figures 3d** and **3e** in the main text, the signal (*i.e.*, the amplitude of the optical phase modulation at the applied frequency) for one pixel in the electro-active area is 6.7 ° for electrical modulation and 0.22 ° without electrical modulation yielding a SNR of ~30 dB.

Furthering the analysis, the distribution over the whole image of the spatio-temporal noise of the system is depicted in **Figure S4a** and corresponds to the standard deviation of the DFT derived amplitude values of the phase modulation at the electrical actuation frequency, for the 16 sets, for each pixel. For the whole image there is a ~ 0.3±0.1 ° average noise level as derived from the associated histogram (**Figure S4b**) that suggests a superposition of the temporal noise with some spatial noise characteristics.

**Figure S4.** Spatio-temporal noise characterization of the ITO-TiO_2_ nanostructured chip analysed with el-epi-MISS: **a.** Standard deviation of DFT phase amplitude at the electrical actuation frequency for each pixel; **b.** Histogram of **a**.

**References**

1. Hofmann, K*. et al*. Properties of evaporated titanium thin films and their possible application in single electron devices. *Thin Solid Films* **436**, 168–174 (2003).

2. Jepsen, O. Electronic structure and magnetic breakdown in titanium. *Physical Review B: Condensed Matter and Materials Physics* **12**, 2988–2997 (1975).

3. Polonschii, C. *et al.* Complementarity of EIS and SPR to Reveal Specific and Nonspecific Binding When Interrogating a Model Bioaffinity Sensor; Perspective Offered by Plasmonic Based EIS. *Analytical Chemistry* **86**, 8553–8562 (2014).

4. Hass, G. & Bradford, A. P. Optical Properties and Oxidation of Evaporated Titanium Films. *Journal of Optical Society America* **47**, 125–129 (1957).

5. Lausmaa, J. Surface spectroscopic characterization of titanium implant materials. *Journal of Electron Spectroscopy and Related Phenomena* **81**, 343–361 (1996).

6. Vaquila, I. *et al.* Chemical reactions at surfaces: titanium oxidation. *Surface Coatings Technology* **122**, 67–71 (1999).

7. Bourdet, P. *et al.* Anodisation of sputtered titanium films: an electrochemical and electrochemical quartz crystal microbalance study. *Thin Solid Films* **483**, 205–210 (2005).

8. Danielsson, P. E. Euclidean distance mapping. Computer Graphics and Image Processing **14**, 227-248 (1980)
